# Preparedness of current and future Saudi Pediatricians to face vaccine hesitancy: Cross-sectional study within the capital city of Saudi Arabia, Riyadh

**DOI:** 10.1016/j.amsu.2021.102718

**Published:** 2021-08-17

**Authors:** Yossef Alnasser, Mahdi A. Alnamnakani, Jawahir M. Abuhaimed, Lulwah Z. Alshiha, Nouf M. Alhamid, Ghada A. Alalshaikh

**Affiliations:** a Pediatric Department, King Saud University Medical City, King Saud University, Riyadh, Saudi Arabia; b Clinical Trails Unit, King Saud University Medical City, King Saud University, Riyadh, Saudi Arabia; c King Saud Medical School, King Saud University, Riyadh, Saudi Arabia

**Keywords:** Vaccine hesitancy, Pediatricians, Knowledge, Attitude, Preparedness

## Abstract

**Background:**

Vaccines have helped in eradicating many communicable diseases. They are considered major players in preserving children's health. However, concerns about vaccines' ingredients and safety became hot topics globally. With doubt, some parents became hesitant to vaccinate their children. A recent study documented high prevalence of vaccine hesitancy among Saudi parents.

**Objectives:**

This study aims to explore preparedness of current and future pediatricians to face vaccine hesitancy, a growing public health issue in Saudi Arabia.

**Methods:**

This study adopted non-interventional cross-sectional online questionnaire specifically designed to encompass general vaccine hesitancy related questions including Covid-19's vaccines.

**Results:**

The study recruited 119 participants form three main tertiary centers in Riyadh. Trainees were the majority with pediatric consultants representing 22%. Females were more than half of total participants (53%). Although familiarity with the term “vaccine hesitancy” was common, it was affected by training level. Among participants, 66% heard about it from social media. Furthermore, only 32% received designated training. Knowledge was suboptimal among all levels except for those who received formal training. Despite 80% encountered vaccine hesitancy, only 55% consider it a common public health issue. Attitude toward vaccine hesitant parents was negative among male physicians (odds ratio of 2.3, P value 0.045). Additionally, majority consider it a form of child neglect (95%). In regard to COVID19's vaccine, 31% were reluctant to get vaccinated themselves.

**Conclusion:**

Pediatric workforce in Saudi Arabia commonly encounters vaccine hesitancy. The strong stand against vaccine hesitant parents might affect rapport with families. Sub-optimal knowledge, negative attitude and emerging COVID19 vaccine hesitancy might negatively impact future efforts. Tailored training and innovative educational platforms are essentials to address vaccine hesitancy in Saudi Arabia.

## Highlights

• This is the first paper we know of that explores readiness to face vaccine hesitancy in Saudi Arabia.
• The paper shows a huge deficit in knowledge regarding addressing vaccine hesitancy.
• The only factor that influenced better knowledge addressing vaccine hesitancy was past medical training.
• Despite high exposure to vacicine hesitancy, only half of participants consider it a common public health issue in Saudi Arabia.
• Intolerance to vaccine hesistant parents is alarming for future confrontational relationships with some families.
• Male physicians carry negative attitude toward vaccine hesitant parents and are less enthusiastic to receive further training to address this issue.
• Female physicians are more likely to be hesitant to get COVID19 vaccine themselves.
• There is a great need for tailored training to address vaccine hesitancy in Saudi Arabia.
• Current and future pediatricians showed interest in using social media as an educational platform.

## 1 Introduction

Vaccines are cornerstones in preventive medicine and public health. They helped in eradicating a lot of communicable diseases and advancing human civilizations [1]. However, vaccines caught bad press with emergence of new diseases. Despite failure to find a link between those diseases and vaccines, lack of other explanations has made the argument linger [2]. Vaccines’ safety came under scrutiny lately, especially when western celebrities took a lead in criticizing it. Around conspiracy theories and fabricated studies, anti-vaccine lobby gradually built their movement. New parents in western world were put in a challenge of deciding to vaccinate their children or not.

Digital globalization and temporary migration might have played a role in planting vaccine hesitancy seeds in Middle East. In a recent study by Alsubaie et al. (2019), vaccine hesitancy was found among 20% of Saudi parents [3]. This alarming number is expected to grow with free Internet access to vast confusing data amid a global pandemic. It will be hard for Saudi parents to decide on vaccinating their children. At the same time, Saudi pediatricians are faced with unprecedented challenge, especially during this trying time of COVID-19 pandemic.

This study aims to explore preparedness and readiness of Saudi future and current pediatricians in addressing growing concerns about vaccine safety in Saudi Arabia. With spread of untrusted resources and rumors through social media, we expect that vaccine hesitancy is going to grow further in Saudi Arabia and might lead to a public health emergency in the near future. We hypothesize that Saudi pediatric healthcare system is not ready to deal with this emerging public health issue. Our hypothesis is based on variable training in dealing with vaccine hesitancy and ineffective communication skills’ training [4].

## 2 Methodology

**Study design:** This study adopted non-interventional cross-sectional method. An online questionnaire specifically designed to encompass general vaccine hesitancy related questions was disseminated through three main tertiary centers within the capital city, Riyadh. Then, data were collected from participants with a ratio of five junior residents to three senior residents and two consultants along with senior registrars. Participants from three main tertiary hospitals (King Saud University Medical City, King Fahad Medical City and King Faisal Specialist Hospital) were contacted. A mass texting messages were sent to all trainees and pediatricians through a texting application. The text provided a link for consent followed by study's questionnaire. Inclusion criteria involved active pediatric trainees, active pediatricians, and willingness to finish study questionnaire. Medical interns and retired physicians were excluded. Upon concluding data collection, data were stored in a private iCloud server with limited accesses controlled by principal investigator. Assuming there are 500 pediatrics trainees and pediatricians in Riyadh, a sample size of 88 would have allowed a power of 80%. To increase study power, authors decided to continue data collection beyond 88 participants until saturation of data and emergence of patterns. Data were collected from November 30, 2020 to April 1, 2021.

**Questionnaire development:** The questionnaire was designed based on literature review of local and international data. The questionnaire aimed to capture six main themes: demographics, knowledge, training, attitude, identified risk factors and stakeholders. Primary language was English to avoid any translation bias especially with formal language of practicing medicine in Saudi Arabia being English. Initially, the questionnaire was evaluated by a pilot study for clearness measures and validity of scoring system. Meanwhile, inclusion and exclusion criteria were finalized.

**Statistical Analysis:** Data were analysed statistically using chi square and one way ANOVA with the help of a contracted biostatistician. P values less or equal to 0.05 were considered statistically significant. Univariate regression analysis was employed to predict factors associated with knowledge and attitude by generating odds ratio.

**Source of** Funding**:** This research did not receive any specific grant from funding agencies in the public, commercial, or non-profit sectors.

**Study Ethics:** Study was according to ethical standard of research ethics committee of King Saud University. It was IRB approved with number E−20-5468 on November 30, 2020. All participants were offered informed consent and allowed to withdraw from the study at any time prior to data analysis. Furthermore, all authors disclose no conflict of interest or financial gains. Reporting of all data was in accordance with the STROCSS criteria [5].Study was registered with research registry number researchregistry6976.

## 3 Results

**Study participants:** Our online based questionnaire attracted 119 participants. Among contributors, 53 participants were junior residents (44%) and 31 (26%) were senior residents. Pediatric senior registrars constituted 8% of participants while pediatric consultants represented 22% (9% trained overseas and 13% trained in Saudi Arabia). Majority of participants had less than 5 years of experience. Gender of partakers was almost equally distributed (53% Females vs 47% Males). Most of the non-trainee participants were affiliated with general pediatrics (33% of total participants). (Table 1).

**Table 1:** Characteristics of study's participants.

		Number	%
Career Status:	Pediatric Junior Resident	53	44
	Pediatric Senior Resident	31	26
	Pediatric Senior Registrar	9	8
	Pediatric Consultant trained overseas	11	9
	Pediatric Consultant trained in Saudi Arabia	15	13
Years of Practice:	Less than 5 years	91	76
	More than 5 years	14	12
	More than 10 years	14	12
Gender	Male	56	47
	Female	63	53
Specialty status	General pediatrician	39	33
	Specialized pediatrician	16	13
	Trainee	64	54

**Fig. 1:**
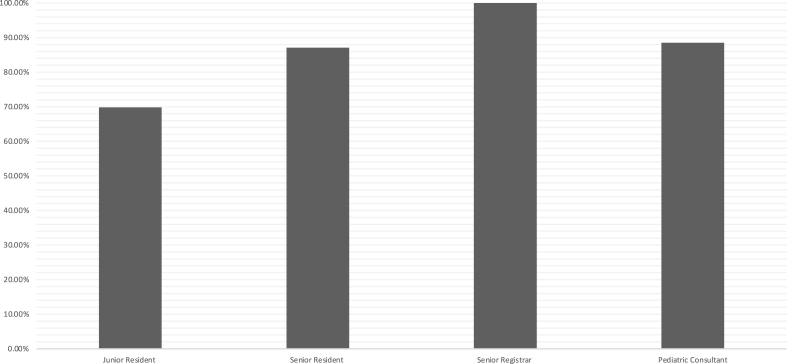
Familiarity with the term “vaccine hesitancy” was noted to be subject to physicians' rank and level of training.

**Knowledge and Experience:** Majority of participants expressed familiarity with the term “vaccine hesitancy” (86%). The familiarity was subject to training level with junior residents expressing least familiarity (70%) which was statistically significant (P value of 0.046) (Fig. 1). Knowledge of how to address vaccine hesitancy was scored arbitrarily with a total of 12 points. Highest score averaged 7.47. Chi square analysis found highest level of knowledge was attributed to previous medical training in addressing vaccine hesitancy (Table 2). Further analysis failed to find any link between training level, location of training, gender, or years of experience with level of knowledge about vaccine hesitancy. Meanwhile, 80% of participants encountered vaccine hesitancy in their practice.

**Table 2:** Means of knowledge scores out of 12 points by different characteristics of study participants.

	Mean	SD	P value
Career status			
Junior Pediatric Residents	6.09	2.36	0.083
Senior Pediatric Residents	7.16	2.16	
Pediatric Registrars	7.44	1.33	
Pediatric Consultants	6.77	1.63	
Years of Practice:			
Less than 5 years	6.54	2.26	0.549
More than 5 years	7.21	1.81	
More than 10 years	6.57	1.60	
Gender			
Male	6.39	1.90	0.274
Female	6.83	2.33	
Specialty			
General pediatricians	6.36	2.24	0.327
Specialized pediatricians	7.31	1.70	
Trainees	6.61	2.17	
Have you received any training on how to address vaccine hesitancy?			
Yes	7.47	1.52	0.001*
No	6.22	2.28	
Saudi trained vs overseas trained			
Pediatric Consultant trained in Saudi Arabia	6.73	1.71	0.899
Pediatric Consultant trained overseas	6.82	1.60	

**Fig. 2:**
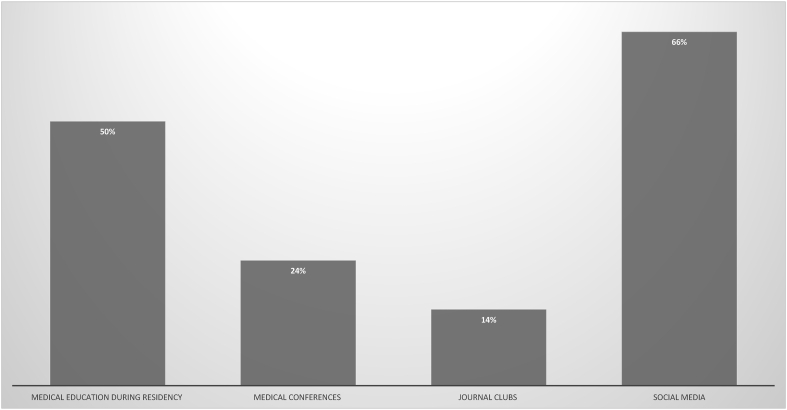
Participants heard about vaccine hesitancy through different outlets with social media being the most popular source of information.

**Education and training:** Despite half of participants (50%) having heard about vaccine hesitancy during residency, only one third received a formal training to address vaccine hesitant parents (32%). Many physicians found information about vaccine hesitancy through social media (66%). Medical conferences presented only 23% of participants with information while journal clubs served only 14% of physicians with information about vaccine hesitancy (Fig. 2). When asked about needing further training, more female physicians were willing to undergo further training to address vaccine hesitancy (92% vs 66%, P value < 0.001).

**Attitude:** Although many participating physicians encountered vaccine hesitancy in their practice, only 55% considered it a common public health issue. Furthermore, the consensus among participants was in favor of mandating vaccination for all children (91%). Actually, the majority considered vaccine hesitancy a form of child neglect (95%). Still, only 12% considered excluding vaccine hesitant parents from their practice. Univariate regression analysis found that negative attitude toward vaccine hesitant parents were higher among male physicians (odds ratio of 2.3, P value 0.045). On the other end, positive attitude was noted among those who were enthusiastic to receive further training (P value = 0.019). When asked about COVID-19 vaccine, 31% of physicians were reluctant to take it themselves. COVID-19 vaccine hesitancy among female physicians was more prominent (P value 0.032).

**Identified Risk Factors and Parental Concern:** Concern about link of vaccines to autism was thought to be a major parental concern by 83% of study participants. When asked about factors influencing vaccine hesitancy, high education was thought to be a major factor by 71% of study participants. It was followed by low education (50%) and low socioeconomic class (25%). Also, parents of children with chronic illnesses were thought to be at risk of vaccine hesitancy by only 21% of physicians. Lowest risk was thought to be associated with returning scholars from developed countries and parents claiming religious causes (17% each). Concerns about pain, safety, and multiple shots were not thought to determine parents’ decision on vaccinating their children. Additionally, Internet and social media were thought to be the biggest influencer of vaccine hesitancy in Saudi Arabia by almost all participants (95%). Lack of trust in healthcare system was thought to be an influencer by one third of physicians (33%).

**Fig. 3:**
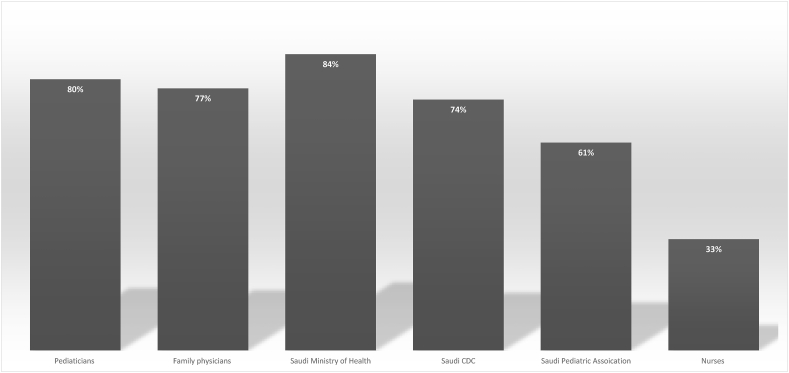
Current and future pediatricians understand the value of their roles in addressing vaccine hesitancy. They expect contributions from ministry of health, Saudi CDC and Saudi Pediatric Association. Least contributions were thought to come from nurses.

**Stakeholders and Leaders:** Majority of participating Saudi pediatricians and pediatric trainees valued their roles in addressing vaccine hesitancy in Saudi Arabia (80%) along with their family physicians colleagues (77%). Majority of participating physicians expect the Saudi Ministry of Health to play a leading role in combating vaccine hesitancy (87%). Most of participating physicians consider Saudi CDC a major stakeholder (74%). Many physicians wanted Saudi Pediatric Association to play an active part in addressing vaccine hesitancy (61%). The least anticipated contributions were thought to be from nurses (33%) **(**Fig. 3**).**

## 4 Discussion

Vaccines have played a major role in sustaining and promoting public health through preventing and eradicating many communicable diseases [6]. Although they have been essential to recent human civilization, their safety has had been debated since the 1800s [7]. This debate was deepened recently by Dr. Andrew Wakefield's false claims of linking MMR vaccine to autism in late 1990 and early 2000 [8]. This claim from such a small size study (12 children) was discredited later and Dr. Wakefield himself was found to be a fraud and his medical license revoked [9]. However, the argument about vaccines' safety lingers. A time of uncertainty during a global pandemic, such as currently being experienced, might provide a solid ground to spread more rumors and false information regarding vaccines.

Recently, vaccine hesitancy was documented in Saudi Arabia. Initially, it presented itself in form of resistance to seasonal influenza vaccine in 2016 [10]. Alabbad et al. [10]found 17% of their study population to be vaccine hesitant. Lack of need and concerns about safety were the most recognized reasons. This report sparked concern about routine childhood vaccine hesitancy which was investigated later in 2018. Alsubaie et al. documented high prevalence of vaccine hesitancy among Saudi parents (20%) [11]. Utilizing WHO SAGE working group on vaccine hesitancy survey tool, they found many educated parents voicing concerns about vaccine safety and link to autism. The latter reasoning mirrored participants' anticipated concern in this study. Also, our findings identified educated parents to be at high risk of vaccine hesitancy echoing Alsubaie et al. (2019) results. However, our findings failed to match previously reported Saudi parents’ concerns about vaccine safety.

Knowledge was sub-optimal in our study, except for those who received formal medical training. Low knowledge might indicate unpreparedness of many current and future pediatricians to face growing vaccine hesitancy. This is not unique to Saudi Arabia as it was reported in other countries [12]. Low knowledge usually accompanies negative attitude [13]. Physicians' negative attitude has been linked to lower vaccination rates in their communities [14]. Physicians’ knowledge and attitude are two determinant factors driving efforts in addressing vaccine hesitant parents. Our study showed male physicians holding negative attitude toward vaccine hesitant parents and having less enthusiasm to receive future training. This makes them a target population in future efforts to tackle vaccine hesitancy in Saudi Arabia.

This paper presents a call for action to provide a proper medical training to prepare current and future Saudi pediatricians to tackle vaccine hesitancy. Such training should start at early stages of medical education [15]. The training should address cultural sensitivity and be tailored to meet local settings [16]. Medical school, residency programs and local conferences need to start adding vaccine hesitancy training to their agenda. Our findings suggested interest from current and future Saudi pediatricians to utilize social media as a source of information. Social media have been used to disseminate information regarding vaccines with good acceptance [17]. With caution and good quality, social media can be implemented as cheap, accessible and widely distributed educational tool [18].

The strong stand against vaccine hesitant parents is quite alarming. Combining less ideal physicians' communication skills [19] and lack of training on addressing vaccine hesitancy, future confrontations and decreased rapport would be expected. Fortunately, Saudi public trust their healthcare system about vaccines’ related information [10]. Also, most of physicians were against excluding vaccine hesitant parents from their practice. Building on trust, keeping families in practices and proper training, vaccine hesitancy can be addressed and prevented in Saudi Arabia.

COVID19 pandemic has reshaped many different aspects of our lives. The pandemic has shifted discussion and attitude about vaccine hesitancy among healthcare providers [20]. Even dialogues in social media about vaccines were shadowed by false information related to the pandemic [21]. Moreover, the pandemic introduced vaccine hesitancy concept to Saudi healthcare providers toward COVID-19 vaccines. Similar to our findings, COVID19 vaccine hesitancy among healthcare providers was reported to be higher among female physicians [22]. Female physicians in Saudi Arabia should be educated about safety and efficacy of COVID19 vaccines. Additionally, the impact of COVID19 pandemic on routine vaccine hesitancy among healthcare providers and Saudi public needs further research.

## 5 Conclusion

Current and future Saudi pediatricians have sub-optimal knowledge on how to address vaccine hesitancy. Male physicians hold negative attitude towards vaccine hesitant parents and are less enthusiastic to learn more on how to address the issue. In contrast, female physicians are more likely to hesitate in getting COVID-19 vaccine themselves. This paper calls for further tailored training to address vaccine hesitancy and strong stand against vaccine hesitant parents to avoid future confrontations and less ideal patient-doctor relationships. Current and future pediatricians are interested in using social media as a source of information which can provide a cheap, accessible and widely distributed educational platform.

### Study limitations and strengths

The study main strength is novelty in our part of the world. This original article provides insights into a growing public health issue. It can be used in designing public health interventions. However, the study geographic limitation might affect its generalizability. More research needs to include primary and secondary healthcare facilities at urban and rural areas along with surveying primary care providers.

## Provenance and peer review

Not commissioned, externally peer-reviewed.

## Please state any conflicts of interest

All authors disclose no conflict of interest.

## Please state any sources of funding for your research

This study received no funding from any governmental, commercial or non-profit organization.

## Ethical approval

This study was IRB approved from King Saud University research ethics committee with a number of E−20-5468.

## Consent

All participants were provided informed consents and allowed to withdraw any time prior to data analysis.

## Author contribution

Authors testify that all persons designated as authors qualify for authorship and have checked the article for plagiarism. YA conceptualized study design, wrote proposal, analysed data and prepared manuscript. MAA optimized study design, edited study proposal, analysed data and edited manuscript. JMA run study logistics, pilot study, data analysis and editing manuscript. LZA helped in study logistics, pilot study, data analysis and writing manuscript. NMA optimized study design, collected data and helped in data analysis. GAA optimized study design, collected data and helped in data analysis.

## Registration of research studies

Name of the registry: Research Registry
Unique Identifying number or registration ID: researchregistry6976
Hyperlink to your specific registration (must be publicly accessible and will be checked): https://www.researchregistry.com/browse-the-registry#home/

## Guarantor

Yossef Alnasser, MD MS

yossef.alnasser@gmail.com.
